# K Fertilizers Reduce the Accumulation of Cd in *Panax notoginseng* (Burk.) F.H. by Improving the Quality of the Microbial Community

**DOI:** 10.3389/fpls.2020.00888

**Published:** 2020-06-26

**Authors:** Yue Shi, Lisha Qiu, Lanping Guo, Jinhui Man, Bingpeng Shang, Rongfeng Pu, Xiaohong Ou, Chunyan Dai, Pengfei Liu, Ye Yang, Xiuming Cui

**Affiliations:** ^1^Yunnan Provincial Key Laboratory of Panax notoginseng, Key Laboratory of Panax notoginseng Resources Sustainable Development and Utilization of State Administration of Traditional Chinese Medicine, Kunming Key Laboratory of Sustainable Development and Utilization of Famous-Region Drug, Faculty of Life Science and Technology, Kunming University of Science and Technology, Kunming, China; ^2^College of Chinese Materia Medica, Beijing University of Chinese Medicine, Beijing, China; ^3^Analysis and Test Center, Kunming University of Science and Technology, Kunming, China; ^4^Chinese Medica Resources Center, China Academy of Chinese Medicinal Sciences, Beijing, China

**Keywords:** bioavailable, cadmium, *Panax notoginseng*, potassium, soil microorganism

## Abstract

The high background value of cadmium (Cd) in the *Panax notoginseng* planting soil is the main reason for the Cd content in *P. notoginseng* exceeding the limit standards. The main goal of this study was to reveal the mechanism by which potassium (K) reduces Cd accumulation in *P. notoginseng* from the perspective of the influences of soil microbial communities on soil pH, total organic matter (TOM) and cation exchange capacity (CEC). Pot experiments were conducted to study the effects of different types and amounts of applied K on the Cd content in *P. notoginseng*, and on the soil pH, TOM, CEC, and bioavailable Cd (bio-Cd) content in soil. Field experiments were conducted to study the effects of K_2_SO_4_ fertilizer on the microbial community, and its correlations with the soil pH, TOM and CEC were analyzed. A moderate application of K_2_SO_4_ (0.6 g⋅kg^–1^) was found to be the most optimal treatment for the reduction of Cd in the pot experiments. The field experiments proved that K fertilizer (K_2_SO_4_) alleviated the decreases in pH, TOM and CEC, and reduced the content of bio-Cd in the soil. The application of K fertilizer inhibited the growth of Acidobacteria, but the abundances of Mortierellomycota, Proteobacteria and Bacteroidetes were promoted. The relative abundances of Acidobacteria and Proteobacteria in the soil bacteria exhibited significant negative and positive correlations with pH and CEC, respectively. In contrast, the relative abundance of Mortierellomycota was found to be positively correlated with the pH, TOM and CEC. The bio-Cd content was also found to be positively correlated with the relative abundance of Acidobacteriia but negatively correlated with the relative abundances of Proteobacteria and Mortierellomycota. The application of K fertilizer inhibited the abundance of Acidobacteria, which alleviated the acidification of the soil pH and CEC, and promoted increase in the abundances of Mortierellomycota, Proteobacteria and Bacteroidetes, which ultimately increased the soil TOM and CEC. Soil microorganisms were found to mitigated decreases in the soil pH, TOM, and CEC and reduced the bio-Cd content in the soil, which significantly reduced the accumulation of Cd in *P. notoginseng*.

## Highlights

– K fertilizers can change the composition and abundance of microbial community in soil.
– Soil microorganisms were found to mitigated decreases in the soil pH, TOM, and CEC.
– The bio-Cd content can be reduced by improving the soil pH, TOM, and CEC.

## Introduction

The genuine producing area of *Panax notoginseng* (Burk.) F. H. is Yunnan Province, China (Yang et al., 2018), which generates approximately 98% of the *P. notoginseng* medicinal materials on the Chinese market (Liu, 2019). However, Yunnan Province accounts for 46% of China’s Cd production (Liu et al., 2016). Its resulted in exceeding the standard rate by 35% and 23% of *P. notoginseng* planting soils and medicinal material, respectively (Ou et al., 2016; Shi et al., 2019). The ability to protect *P. notoginseng* from Cd has drawn considerable attention from consumers and regulatory departments (Lin et al., 2014). Thus, there is a need to develop a low-cost and high-efficiency Cd-blocking technology for *P. notoginseng* as well as to elucidate the underlying mechanisms by which Cd can be blocked.

Lowering the bioavailable Cd (bio-Cd) content in the soil is currently the main method for the reduction of the amount of Cd absorbed by plants (Guiwei et al., 2010). The soil pH, organic matter, cation exchange capacity (CEC) and other soil physical and chemical properties strongly influence on the bioavailability of heavy metals in the soil (Yuan, 2014) and thus affecting the migration of heavy metals from soil to crops (Huang et al., 2012). An increase in pH leads to a corresponding rise in OH^–^ levels and improves the ability of oxide colloids to adsorb and bind heavy metals. As a result, the soil adsorptive capacity for Cd^2+^ increases, thereby increasing the amount of Cd precipitation in the soil (Ardestani and Van Gestel, 2013; Hong et al., 2014). As the soil CEC increases, the soil’s adsorption and retention of heavy metal cations increases, and its specific adsorption of anions weakens, resulting in a decrease in the bioavailability of heavy metals (e.g., Cd, Pb, Hg) in the soil (Chen et al., 2018). An increase in total organic matter (TOM) can increase pH in the soil and the solid organic matter adsorption of heavy metals (Belay et al., 2002). These changes can also decrease the exchangeable heavy metal content (Zeng et al., 2011).

Soil microorganisms can decompose organic matter and alter the TOM content (Neumann et al., 2014). Yang et al. (2011) found that bacterial biomass in orchard soil exhibited a significant positive correlation with soil organic matter. Soil microorganisms also significantly interacted with pH. Sait et al. (2006) found a significant negative correlation between colonial development in Acidobacteria and the soil pH. The number of soil fungi interacted with the soil CEC, pH, and available K content, and was significantly positively correlated with the available K content and CEC (Zhang et al., 2011). Therefore, soil microorganisms are an important index for the evaluation of the evaluating soil pH, TOM, and CEC (Sanusi, 2015).

Potassium is often considered as a quality element (Radulov et al., 2014). Simultaneously, the application of K as a fertilizer can reduce the exchangeable lead content in wheat planting soil, thus reducing the inhibition of the increase of the dry weight (Chen et al., 2007a, b). Zhao et al. (2004) found that K_2_SO_4_ fertilizer could decrease the carbonates fraction of Cd [F(Carb)] and the exchangeable fraction of Cd [F(EXC)] in wheat planting soil, resulting in the reduction of the Cd content in wheat. Wang et al. (2017) indicated that KHCO_3_ fertilizer could reduce the Cd content in tobacco and alleviate Cd toxicity during growth. Thus, it is evident that K plays an important role in the reduction of the bio-Cd content in the soil, thereby reducing its accumulation in plants.

Duan et al. (2015) demonstrated that applying an appropriate amount of K fertilizer could also improve the diversity of fungal species in soil by restricting the growth of certain fungi and effectively preventing the over propagation of pathogenic fungi. Jia et al. (2004) proved that K fertilizers promoted the growth of soil microorganisms and contributed to the mineralization of the soil organic matter in buckwheat planting soil. In the present research, it was hypothesized that K fertilization can indirectly improve soil physical and chemical properties indirectly by influencing the soil microorganisms. Consequently, the bio-Cd content was found to be reduced in the soil. This process is a key mechanism for the reduction of the accumulation of Cd in *P. notoginseng* under the application of K fertilization. However, there currently exists no direct evidence to support this hypothesis.

Accordingly, pot experiments and 2-year field experiments were performed to explore the effects of different K fertilizers and application amounts on the soil on pH, CEC, TOM, soil microorganisms, and Cd content in *P. notoginseng*. The amount of K fertilizer applied in the cultivation of *P. notoginseng* was optimized, and soil improvement and utilization were combined to promote the reduction of Cd in *P. notoginseng*.

## Materials and Methods

### Pot Experiments

Pot experiments were conducted from May 5 to September 5 in 2017, and the experimental site was located in faculty of life science and technology of Kunming university of science and technology (E 102.51, N 24.50, altitude 1982 m). Main environment of the greenhouse was as follows: soil moisture, 33–48%; air humidity, 35–82%; daytime temperature, 12–29°C; night temperature, 8–18°C; sunshine duration, 9–11 h.

Two-year-old *P. notoginseng* was planted in the plastic pot (70 × 40 × 28 cm) with 18 kg soil. The soil was the same as that of the greenhouse of tillage layers (0–20 cm). The soil was dried and crushed, separated from weeds, rocks and other debris, and then sifted and reserved. According to the conclusion of Li et al. (2015), 10 mg⋅kg^–1^ Cd treatment was set up. Under the same K treatment level, the amount of K input by different types of K fertilizer was consistent. Simultaneously, KCl and K_2_SO_4_ were adopted, respectively. Three K (K_2_O) levels were used as follows, low application amount: 0.171 g⋅kg^–1^ (KCl1), 0.2 g⋅kg^–1^ (K_2_SO_4_1); medium application amount: 0.513 g⋅kg^–1^ (KCl2), 0.6 g⋅kg^–1^ (K_2_SO_4_2); high application amount: 1.026 g⋅kg^–1^ (KCl3), 1.2 g⋅kg^–1^ (K_2_SO_4_3), respectively. The treatments were as follows: CK (control), Cd, Cd + KCl1, Cd + K_2_SO_4_1, Cd + KCl2, Cd + K_2_SO_4_2, Cd + KCl3, Cd + K_2_SO_4_3.

Eight treatments were conducted, every treatment was repeated three times (three pots), every pot planted eight seedlings. Nitrogen (carbamide) and phosphate (P_2_O_5_) fertilizers were used at 0.30 and 0.10 g⋅kg^–1^, respectively. All of fertilizers were applied as basal fertilizer. Cd was soluted in distilled water, while basal fertilizers were mixed up with dry soil, and then added to the pots.

### Field Experiments

In Qiubei County, Wenshan City, Yunnan Province, field experiments were performed from January to November of 2018 and 2019, respectively (Malishu, E: 103°61′, N: 23°87′, altitude: 1937 m; Longgaxinzhai, E: 104°1′, N: 24°11′, altitude: 1452 m). The basic soil physical and chemical properties of Malishu was as follows: pH, 5.76; TOM, 5.63 g⋅kg^–1^; CEC, 7.33 c mol⋅kg^–1^; total K 24.28 g⋅kg^–1^; total P, 0.52 g⋅kg^–1^; total N, 1.12 g⋅kg^–1^; alkali-hydrolyzed N 54.00 mg⋅kg^–1^; available P 1.15 mg⋅kg^–1^; and available K 83.00 mg⋅kg^–1^. The basic soil physical and chemical properties of Longgaxinzhai was as follows: pH, 5.88; TOM, 5.58 g⋅kg^–1^; CEC, 7.69 c mol⋅kg^–1^; total K 24.71 g⋅kg^–1^; total P, 0.35 g⋅kg^–1^; total N, 1.04 g⋅kg^–1^; alkali-hydrolyzed N 53.20 mg⋅kg^–1^; available P 1.08 mg⋅kg^–1^; and available K 87.00 mg⋅kg^–1^. The planting soil of *P. notoginseng* was red soil.

According to the optimum application type and amount of K fertilizer in the pot experiments, the optimum application amount of K fertilizer (K_2_SO_4_, 300 kg⋅ha^–1^ both in 2018 and 2019) in field experiment were performed according to the soil weight conversion of 20 cm deep plow layer. Simultaneously, the amount of K fertilizer (15 kg⋅ha^–1^ both in 2018 and in 2019) was set as the control. The treatments were as follows: K_15_, K_300_. All groups were repeated three times. The 15 kg⋅ha^–1^ (K_15_) and 300 kg⋅ha^–1^ (K_300_) were used as the application amounts. The 225 kg⋅ha^–1^ was used as the amount of P, N fertilizers. Thirty percent of the N and K fertilizers were applied as base, and 70% were applied as topdressing fertilizers. The topdressing fertilizer were applied at May, June, August and October, respectively, and the application rates were 20, 10, 20, and 20%, respectively. All P fertilizer was used as basal fertilizer. Around the experimental area, a protective row (width, 1 m) was set up to protect the performance of the experiments. The plot area was 2.30 m × 1.90 m, the transplant density was 15 cm × 15 cm, and seedlings were transplanted in January 2018 and 2019, respectively. In November 2018 and 2019, *P. notoginseng* and soil samples were collected. Field management was performed according to farmers’ customary management.

### Determination of Cd Content

#### Determination of Cd Content in *P. notoginseng*

According to Shi et al. (2019), microwave digestion with HNO_3_-H_2_O_2_ was used to digest the Cd content in *P. notoginseng*. Dried sample (0.20 g) was accurately weighed (accurate to 0.0001 g) and placed it in the Teflon dissolving cup. Then, 10 ml 65% HNO_3_ was added, and left overnight for pre-reaction and when 2 ml 30% H_2_O_2_ was added. Until the reaction was stable, the sample cup was covered, placed it in a high-pressure tank, and then putting into a microwave sample dissolving device. The step temperature increased to 180°C for 25 min (the power of the single tank was 600 w). While the sample was dissolved, the temperature was reduced to room temperature. The sample was transferred to a 10 ml volumetric flask, water was used to scale, and the sample was shaken well. And a blank control was made at the same time. The Cd content was determined by inductively coupled plasma atomic emission spectrometry (ICP-AES).

#### Speciation of Cd in the Soil and Determination of Cd Content

The soil Cd classification adopted the improved Tessier A five-step extraction method (Tessier et al., 1979). Soil was divided into the following five components:

F(EXC): The sediment was continuously extracted for 1 h with 8 ml 1 M MgCl_2_ solution (pH = 7.00); then, centrifuged at 4000 r min^–1^ for 10 min and filled to constant volume to be measured.

F(Carb): The residue from previous was continuously extracted for 5 h with 8 ml 1 M NaAc solution (pH = 5.00), then 4000 r min^–1^ for 10 min centrifuged and made it constant volume to be measured.

F(Fe-MnOX): The residue from previous was continuously extracted for 6 h with 25% HAC solution of 20 ml 0.04 M NH_2_OH⋅HCl at 96 ± 3°C; then, centrifuged at 4000 r min^–1^ for 10 min and filled to constant volume to be measured.

F(OM): The residue from previous was continuously extracted for 2 h with 3 ml 0.02 M HNO_3_ and 5 ml 30% H_2_O_2_ solution (pH = 2.00) at 85 ± 3°C, continuously extracted for 3 h with 3 ml 30% H_2_O_2_, and cooled to room temperature; then, continuously extracted for 30 min with 5 ml 20% HNO_3_ of 3.20 M NH_4_AC; then, centrifuged at 4000 r min^–1^ for 10 min and filled to constant volume to be measured.

F(RES): The residue from previous was digested with HF-HClO_4_.

Cd was analyzed using inductively coupled plasma mass spectroscopy (ICP-MS, X Series 2, Thermo Fisher Scientific, United States).

### Determination of Soil pH, TOM, and CEC

Soil pH determination method refers to ISO 10390:2005 standard, which determined with a pH meter (FE20, Mettler, China) after mixing soil and water at a 1–2.5 ratio.

TOM determination method refers to ISO 23470–2011 standard, 0.10–0.50 g (0.001 g) soil sample was weighed, then mixed with 0.10 g AgSO_4_, 5 ml 0.80 M K_2_Cr_2_O_7_ solution and 5 ml H_2_SO_4_, then heated to 180°C for 5 min, and titrated with 0.20 M (NH_4_)_2_ Fe (SO_4_)_2_ standard solution.

CEC determination method refers to ISO 14235–2009 standard, 2.00 g soil sample was weighed, mixed with 60 ml 1 M ammonium acetate solution, centrifuged at 3000 r min^–1^ for 5 min, centrifugation was repeated until supernatant had no calcium ions. Then, 60 ml 95% ethanol was added and centrifuged, the above steps were repeated until the supernatant had no ammonium ions. The solution was distilled by Kjeldahl apparatus and titrated with HCl standard solution.

### Determination of Microbial Diversity and Population Composition

The experimental samples were taken from the above *P. notoginseng* planting soil in field experiments. Total bacterial and fungal DNA were extracted from samples using the Power Soil DNA Isolation Kit (MO BIO Laboratories) according to the manufacturer’s protocol. DNA quality and quantity were assessed by the ratios of 260 nm/280 nm and 260 nm/230 nm. Then DNA was stored at −80°C until further processing. The V3-V4 region of the bacterial 16S rRNA gene was amplified with the common primer pair (Forward primer, 5′-ACTCCTACGGGAGGCAGCA-3′; reverse primer, 5′-GGACTACHVGGGTWTCTAAT-3′) combined with adapter sequences and barcode sequences. The fungal ITS rRNA gene was amplified with the common primer pair (Forward primer, 5′-CTTGGTCATTTAGAGGAAGTAA-3′; reverse primer, 5′-GCTGCGTTCTTCATCGATGC-3′) combined with adapter sequences and barcode sequences. PCR amplification was performed in a total volume of 50 μl, which contained 10 μl Buffer, 0.2 μl Q5 High-Fidelity DNA Polymerase, 10 μl High GC Enhancer, 1 μl dNTP, 10 μM of each primer and 60 ng genome DNA. Thermal cycling conditions were as follows: an initial denaturation at 95°C for 5 min, followed by 15 cycles at 95°C for 1 min, 50°C for 1 min and 72°C for 1 min, with a final extension at 72°C for 7 min. The PCR products from the first step PCR were purified through VAHTSTM DNA Clean Beads. A second round PCR was then performed in a 40 μl reaction which contained 20 μl 2 × Phusion HF MM, 8 μl ddH_2_O, 10 μM of each primer and 10 μl PCR products from the first step. Thermal cycling conditions were as follows: an initial denaturation at 98°C for 30 s, followed by 10 cycles at 98°C for 10 s, 65°C for 30 s and 72°C for 30 s, with a final extension at 72°C for 5 min. Finally, all PCR products were quantified by Quant-iT^TM^ dsDNA HS Reagent and pooled together. High-throughput sequencing analysis of bacterial and fungal rRNA genes were performed on the purified, pooled sample using the Illumina Hiseq 2500 platform (2 × 250 paired ends) at Biomarker Technologies Corporation, Beijing, China.

### Statistical Analysis

Data were processed with Microsoft Excel software 2018, Graphpad Prism 7.0 SPSS 24.0 were applied to fit the curves and analyze statistics, respectively. Duncan’s multiple range tests of one-way ANOVA were used to analyze data for separating means. When *P* < 0.05, differences were considered significant. The Spearman correlation analysis was used to assess the association of the relative abundance of microbial community with pH, TOM, CEC, and bio-Cd.

## Results

### Effect of K Fertilizer on Cd Content in *P. notoginseng* in the Pot Experiments

**FIGURE 1:**
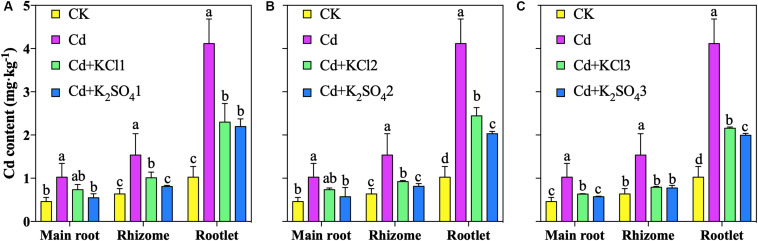
Effect of different types of K on Cd content in *P. notoginseng* (**A** denoted low application amount, KCl1: 0.171, K_2_SO_4_1: 0.2 g⋅kg^–1^; **B** denoted medium application amount, KCl2: 0.513, K_2_SO_4_2: 0.6 g⋅kg^–1^; **C** denoted high application amount, KCl3: 1.026, K_2_SO_4_3: 1.2 g⋅kg^–1^). Different lowercase letters indicate the means are significantly different at *P* < 0.05.

The results of the pot experiment demonstrated that the Cd accumulation in *P. notoginseng* roots decreased under different types of K fertilizer treatments (Figure 1). Relative to that of Cd the treatment, the Cd content in the main root under the KCl2 and K_2_SO_4_2 treatments decreased by 28 and 44%, respectively; the Cd content in the rhizome decreased by 40 and 47%, respectively; and the Cd content in the rootlets decreased by 41 and 51%, respectively. The K_2_SO_4_ treatment resulted in the largest reduction in Cd accumulation and was thus adopted in the subsequent experimental treatments.

Relative to that under the Cd treatment, the Cd content in the main root under the K_2_SO_4_1, K_2_SO_4_2, and K_2_SO_4_3 treatments decreased by 46, 44, and 44%, respectively; that in the rhizome decreased by 47, 47, and 50%, respectively; that in the rootlets decreased by 47, 51, and 52%, respectively (Figure 1). The reduction in the accumulation of Cd in *P. notoginseng* under the moderate K fertilization treatment was similar to that under the high K fertilization treatment. Therefore, 0.6 g⋅kg^–1^ K_2_SO_4_ was converted into an application of 300 kg⋅ha^–1^ for the subsequent field verification experiment.

### Effect of K Fertilizer on the pH, TOM, and CEC of *P. notoginseng* Planting Soil in the Pot Experiments

**FIGURE 2:**
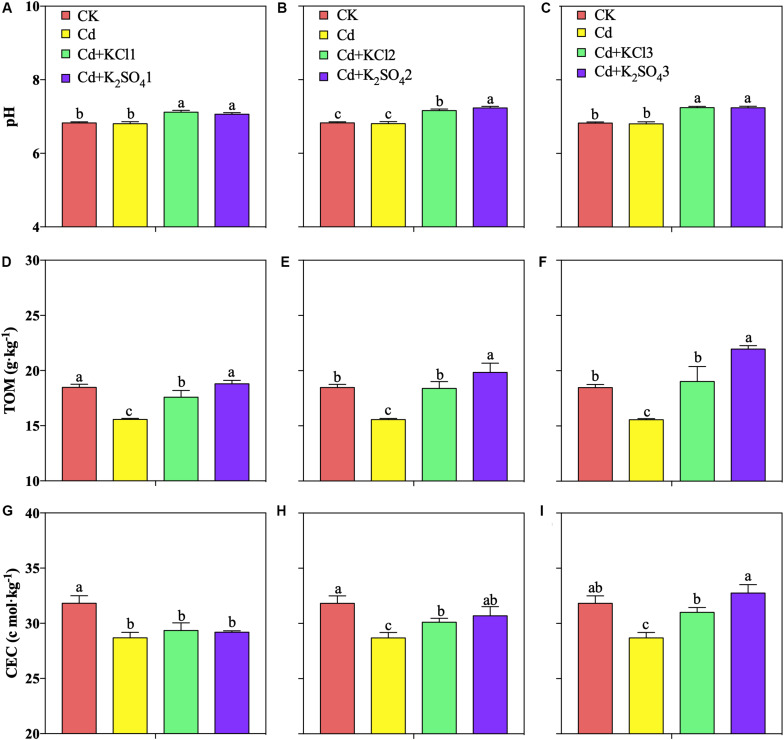
Effect of different types and application amounts of K on pH, TOM, and CEC. **(A,D,G)** Denoted low application amount, KCl1:0.171, K_2_SO_4_1:0.2 g⋅kg^–1^; **(B,E,H)** denoted medium application amount, KCl2:0.513, K_2_SO_4_2:0.6 g⋅kg^–1^; **(C,F,I)** denoted high application amount, KCl3:1.026, K_2_SO_4_3:1.2 g⋅kg^–1^. Different lowercase letters indicate the means are significantly different at *P* < 0.05.

In the pot experiments, low K fertilizer treatments significantly improved the soil pH and TOM, but different types of K fertilizer treatments did not significantly affect the soil pH (Figures 2A,D,G). A moderate amount of K fertilizer was found to significantly promote the increase of the soil pH, TOM, and CEC. The pH levels of the soil under the KCl2 and K_2_SO_4_2 treatments increased by 5 and 6%, respectively; the TOM increased by 18 and 27%, respectively; and the CEC increased by 5 and 7%, respectively (Figures 2C,E,H).

### Effect of K Fertilizer on the Bio-Cd Content in *P. notoginseng* Planting Soil in the Pot Experiments

**FIGURE 3:**
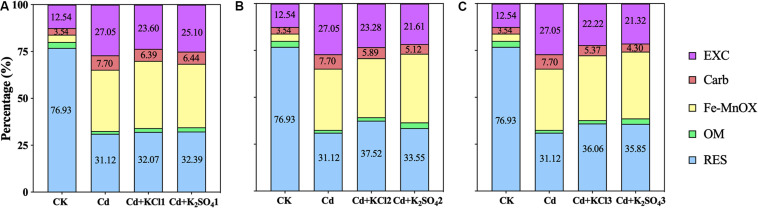
Effect of different types and application amounts of K on Bio-Cd content in *P. notoginseng* planting soil **(A)** denoted low application amount, KCl1: 0.171, K_2_SO_4_1: 0.2 g⋅kg^–1^; **(B)** denoted medium application amount, KCl2: 0.513, K_2_SO_4_2: 0.6 g⋅kg^–1^; **(C)** denoted high application amount, KCl3: 1.026, K_2_SO_4_3: 1.2 g⋅kg^–1^. Bio-Cd content is the sum of F(EXC) and F(Carb) Cd.

Compared with the Cd treatment, all K fertilizer treatments reduced the soil bio-Cd content in the soil (Figure 3). When a moderate amount of K fertilizer was applied, the bio-Cd contents under the KCl2 and K_2_SO_4_2 treatments decreased by 16 and 23%, respectively.

### Effect of K_2_SO_4_ on Cd Accumulation in *P. notoginseng* in the Field Experiments

**FIGURE 4:**
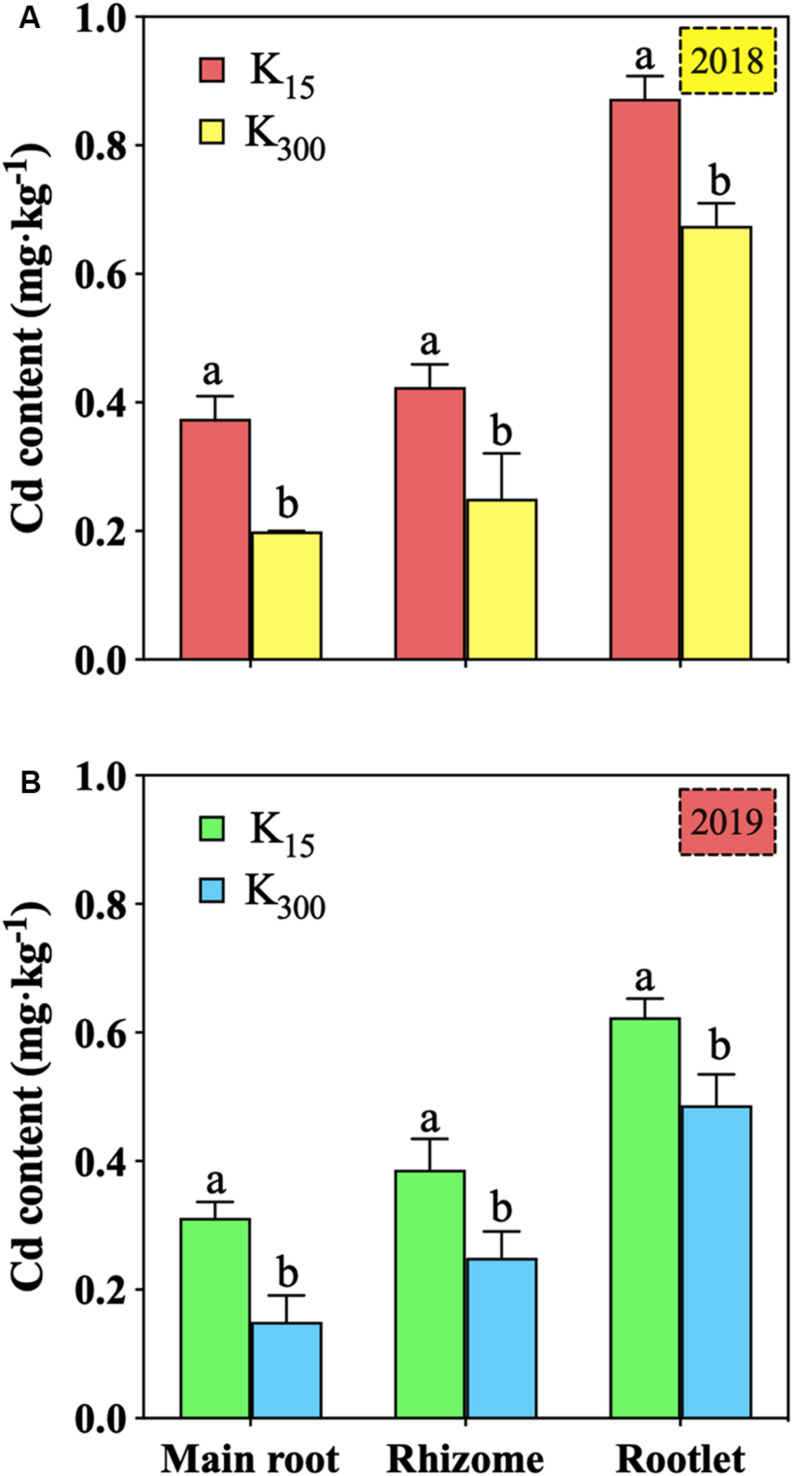
Effect of different amounts of K_2_SO_4_ on the Cd content in *P. notoginseng* roots (K_15_, 15 kg⋅ha^–1^; K_300_, 300 kg⋅ha^–1^; **(A)** denoted experiment was conducted in 2018, **(B)** denoted experiment was conducted in 2019). Different lowercase letters indicate the means are significantly different at *P* < 0.05.

Relative to that under the K_15_ treatment, the Cd content in the main root under the K_300_ treatment decreased by 47%, that in the rhizome decreased by 41%, and that in the rootlets decreased by 23% in 2018; additionally, the Cd content in the main root decreased by 52% in 2019 (Figure 4). Regarding the K_300_ treatments in 2018 and 2019, the Cd content in the main root and rhizome were 0.20 (2018), 0.15 (2019) and 0.25 (2018), 0.25 (2019) mg⋅kg^–1^, respectively, both of which were within the WM-T2-2004 standard (WM-T2-2004, 2004) (0.3 g⋅kg^–1^).

### Effect of K_2_SO_4_ on the pH, TOM, and CEC of *P. notoginseng* Planting Soil in the Field Experiments

**FIGURE 5:**
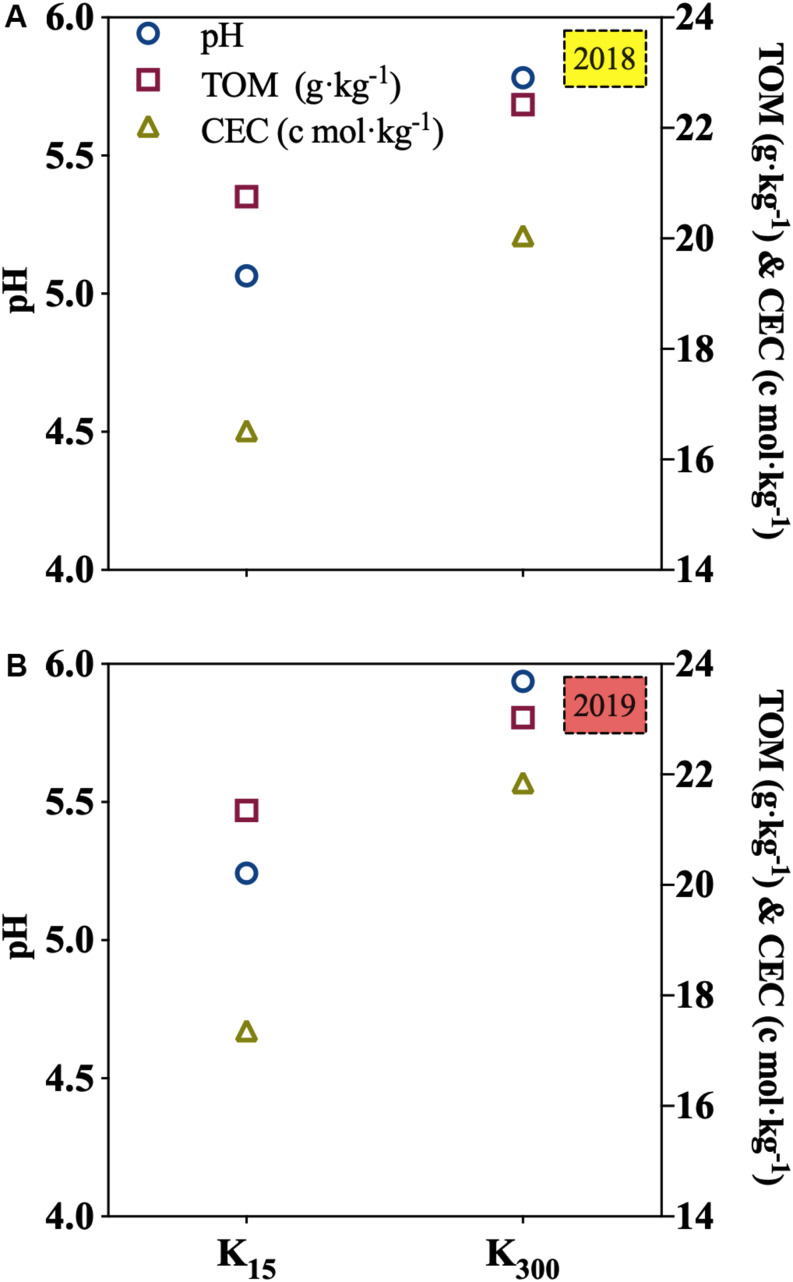
Effect of different amounts of K_2_SO_4_ on pH, TOM and CEC in soil (K_15_, 15 kg⋅ha^–1^; K_300_, 300 kg⋅ha^–1^; **(A)** denoted experiment was conducted in 2018, **(B)** denoted experiment was conducted in 2019. Different lowercase letters indicate the means are significantly different at *P* < 0.05.

Relative to those under the K_15_ treatment, the pH, TOM, and CEC of the soil under K_300_ treatment were increased by 14, 8, and 21%, respectively, in 2018; in 2019, the values of these increments were 13, 8, and 26%, respectively (Figure 5).

### Effect of K_2_SO_4_ on Microbial Community Composition in *P. notoginseng* Planting Soil in the Field Experiments

**FIGURE 6:**
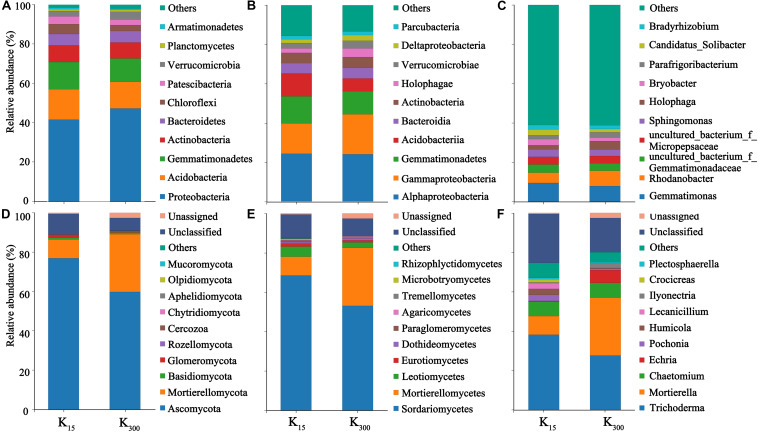
Effect of different amounts of K_2_SO_4_ on the community composition and relative abundance of Bacteria (**A**, Phylum; **B**, Class; **C**, Genus), Fungi (**D**, Phylum; **E**, Class; **F**, Genus) in *P. notoginseng* planting soil in 2018 (K_15_, 15 kg⋅ha^–1^; K_300_, 300 kg⋅ha^–1^).

**FIGURE 7:**
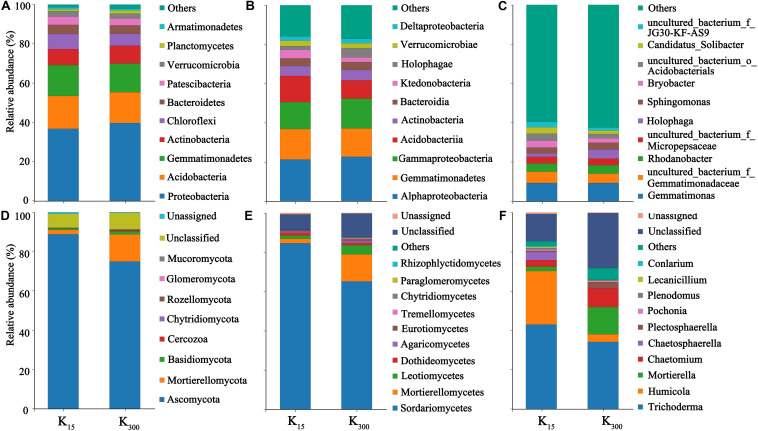
Effect of different amounts of K_2_SO_4_ on the community composition and relative abundance of Bacteria (**A**, Phylum; **B**, Class; **C**, Genus), Fungi (**D**, Phylum; **E**, Class; **F**, Genus) in *P. notoginseng* planting soil in 2018 (K_15_, 15 kg⋅ha^–1^; K_300_, 300 kg⋅ha^–1^).

The sequences were submitted to the SRA (Sequence Read Archive) at the National Center for Biotechnology Information (NCBI) under the accession number PRJNA626539 for 16S sequences (B1–B12) and ITS sequences (F1–F12). At the phylum level, 25 bacterial phyla and 10 fungal phyla were detected in eight samples under the two treatments. The bacterial communities in all treated samples primarily consisted of Proteobacteria, Acidobacteria, Gemmatimonadetes, Actinobacteria, Bacteroidetes, and other dominant phylum-level species with relative abundances of more than 5%. The fungal communities were primarily composed of Ascomycota, Mortierellomycota, and other dominant phylum-level species. The relative abundances of Proteobacteria and Verrucomicrobia increased under the K_2_SO_4_ treatment. The relative abundances of Proteobacteria significantly increased by 12% (2018) and 7% (2019), those of Acidobacteria significantly decreased by 13% (2018) and 6% (2019); and those of Chloroflexi significantly decreased by 17% (2018) and 21% (2019) (Figures 6A, 7A). The relative abundances of Mortierellomycota significantly increased by 208% (2018) and 513% (2019), whereas those of Ascomycota significantly decreased by 22% (2018) and 21% (2019) (Figures 6D, 7D).

Moreover, 65 bacterial classes and 23 fungal classes were detected in this study. The relative abundances of Acidobacteria decreased by 45% (2018) and 30% (2019) with the application of K_2_SO_4_ (Figures 6B, 7B). The relative abundances of Mortierellomycetes increased by 208% (2018) and 515% (2019), whereas those of Sordariomycetes significantly decreased by 22% (2018) and 23% (2019) (Figures 6E, 7E). A total of 348 bacterial genera (Figures 6C, 7C) and 118 fungal genera (Figures 6F, 7F)were detected in this study.

### Correlation Analysis of Microbial Community Composition, pH, TOM, and CEC of *P. notoginseng* Planting Soil Under Different K_2_SO_4_ Treatments in the Field Experiments

At the phylum level, the relative abundances of Proteobacteria and Planctomycetes in the soil bacteria showed significant positive correlations with the pH and CEC, whereas the relative abundances of Chloroflexi exhibited a negative correlation with pH and CEC in both 2018 and 2019 (Table 1). The relative abundance of Mortierellomycota was found to exhibit significant positive correlations with the pH and CEC in the soil, whereas the relative abundance of Ascomycota exhibited a negative correlations with the pH, TOM, and CEC in the soil in 2018 and 2019.

**TABLE 1:** Correlation analysis of soil microbial communities at the phylum level and soil physical and chemical properties in *P. notoginsen*g planting soil under different K_2_SO_4_ treatments.

	Bacterial				Fungal		
Community phylum	pH	TOM	CEC	Community phylum	pH	TOM	CEC
**2018**							
Proteobacteria	0.786*	0.857**	0.762*	Ascomycota	−0.833**	−0.667*	−0.857**
Acidobacteria	–0.095	0.214	–0.71	Mortierellomycota	0.714*	0.810	0.738*
Chloroflexi	−0.762*	–0.357	−0.738*	Chytridiomycota	–0.595	−0.810*	–0.643
Patescibacteria	–0.595	−0.810*	–0.571	–	–	–	–
Verrucomicrobia	0.786*	0.310	0.762*	–	–	–	–
**2019**							
Proteobacteria	0.935**	0.907**	0.882**	Ascomycota	−0.923**	−0.895**	−0.870**
Acidobacteria	–0.525	–0.509	–0.495	Mortierellomycota	0.970**	0.941**	0.914**
Chloroflexi	−0.987**	−0.948**	−0.922**	Basidiomycota	0.893**	0.866**	0.842**
Actinobacteria	0.934**	0.906**	0.881**	Rozellomycota	0.994**	0.964**	0.937**
Armatimonadetes	−0.763*	−0.741*	−0.720*	Glomeromycota	0.974**	0.945**	0.918**
Planctomycetes	0.823*	0.798*	0.776*	–	–	–	–

*Shows the correlation reached P < 0.05, **shows the correlation reached P < 0.01. TOM, total organic matter; CEC, cation exchange capacity.

At the class level, the relative abundance of Acidobacteriia in the soil bacteria was negatively correlated with the pH and CEC in 2018 and 2019. Moreover, the relative abundances of Verrucomicrobiae (2018) and Alphaproteobacteria (2018, 2019) respectively exhibited significant positive correlations with the pH and CEC, respectively. The relative abundance of Mortierellomycetes was positively correlated with the TOM, whereas the relative abundances of Sordariomycetes (2018, 2019) were negatively correlated with the pH and CEC of the soil (Table 2).

**TABLE 2:** Correlation analysis of soil microbial communities at the class level and soil physical and chemical properties in *P. notoginsen*g planting soil under different K_2_SO_4_ treatments.

	Bacterial				Fungal		
Community class	pH	TOM	CEC	Community class	pH	TOM	CEC
**2018**							
Alphaproteobacteria	0.500	0.095	0.548	Sordariomycetes	−0.833**	–0.667	−0.857**
Acidobacteriia	−0.905**	–0.643	−0.881**	Mortierellomycetes	0.714*	0.810*	0.738*
Verrucomicrobiae	0.786*	0.310	0.762*	Leotiomycetes	−0.810*	−0.738*	−0.762*
Deltaproteobacteria	0.881**	0.571	0.857**	–	–	–	–
**2019**							
Alphaproteobacteria	0.868**	0.842**	0.818*	Sordariomycetes	−0.930**	−0.902**	−0.877**
Acidobacteriia	−0.955**	−0.926**	−0.900*	Mortierellomycetes	0.970**	0.941**	0.914**
Actinobacteria	0.835**	0.810*	0.787*	Eurotiomycetes	0.940**	0.912**	0.886**
Gemmatimonadetes	−0.924*	−0.897*	−0.871*	–	–	–	–
Gammaproteobacteria	0.927*	0.899*	0.784*	–	–	–	–

*Shows the correlation reached P < 0.05, **shows the correlation reached P < 0.01. TOM, total organic matter; CEC, cation exchange capacity.

**FIGURE 8:**
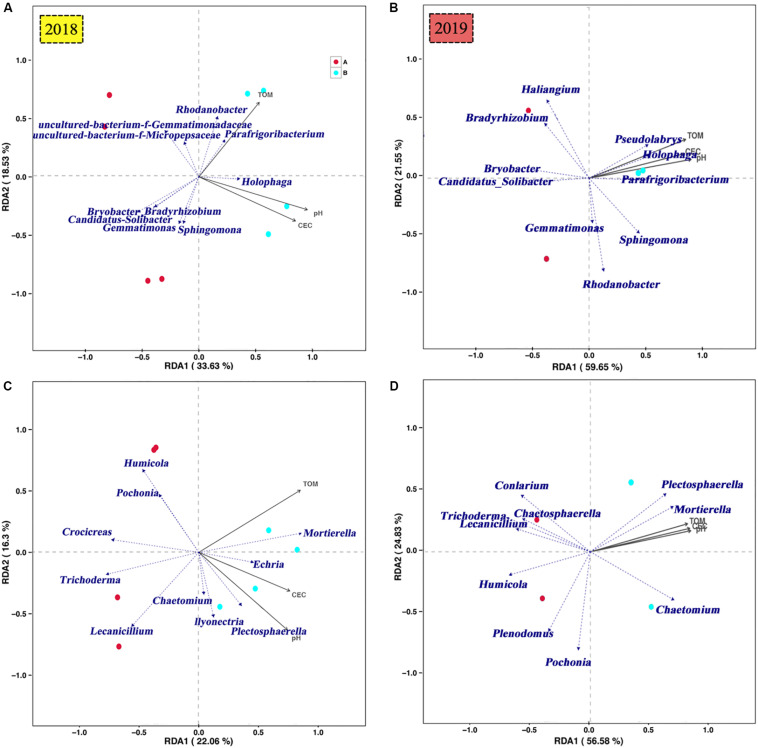
RDA analysis of soil microbial community [**A**, bacteria (2018); **B**, fungi (2018); **C**, bacteria (2019); **D**, fungi (2019)] at the genus level and soil pH, TOM, and CEC in *P. notoginsen*g planting soil under different K_2_SO_4_ treatments.

Redundancy analysis of dominant genus-level species of bacteria and the pH, TOM and CEC in *P. notoginseng* planting soil was conducted (Figure 8). The first ordination axis in Figures 8A,C explained 34% (2018) and 59% (2019) of the dominant genus-level species of soil bacteria, whereas the second ordination axis explained 19% (2018) and 22% (2019) of the same. This indicates that the amount of K_2_SO_4_ fertilizer was positively correlated with the pH, TOM, and CEC. The correlation *P*-values of the correlations between Holophaga (2018, 2019), Candidatus_Solibacter (2018), Bradyrhizobium (2018), Pseudolabrys (2019), and TOM were all less than 0.05 (Figure 8), indicating that the relative abundances of the aforementioned communities significantly affected the TOM content of the soil.

The first ordination axis in Figures 8B,D explained 22% (2018) and 57% (2019) of the variation in the dominant genus-level species of soil fungi, and the second ordination axis explained 16% (2018) and 25% (2019). The correlation *P-*values of the correlations between Mortierella (2018, 2019), Humicola (2018), Lecanicillium (2018), and Plectosphaerella (2019), and the pH and CEC were all less than 0.05 (Figure 8), indicating that the relative abundances of these communities significantly affected the pH and CEC of the soil.

### Effect of K_2_SO_4_ on Bio-Cd Content in *P. notoginseng* Planting Soil in the Field Experiments

**FIGURE 9:**
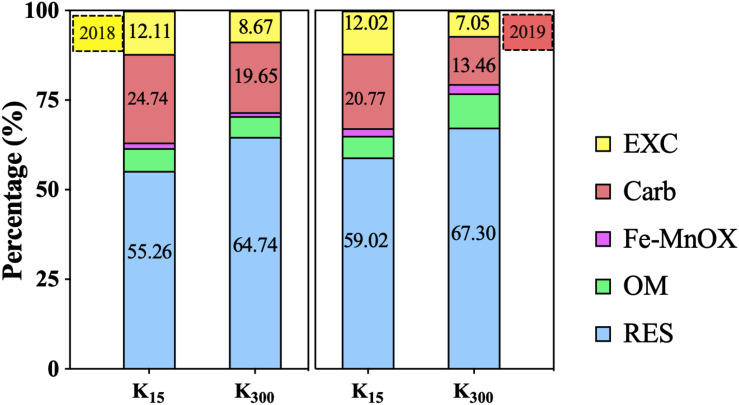
Effect of different amounts of K_2_SO_4_ on Bio-Cd content in *P. notoginseng* planting soil [K_15_, 15 kg⋅ha^–1^; K_300_, 300 kg⋅ha^–1^. Bio-Cd content is the sum of F(EXC) and F(Carb) Cd].

K_2_SO_4_ treatment can significantly decrease the bio-Cd content in *P. notoginseng* planting soil (Figure 9). Relative to that under the K_15_ treatment, the bio-Cd content under the K_300_ treatment decreased by 23% in 2018 and 37% in 2019. The bio-Cd content was found to be negatively correlated with pH, TOM and CEC in *P. notoginseng* planting soil in 2018 and 2019 (Table 3).

**TABLE 3:** Correlation analysis of bioavailable Cd content and soil physical and chemical properties in *P. notoginsen*g planting soil under different K_2_SO_4_ treatments.

	2018	2019
	Bio-Cd content	Bio-Cd content
pH	−0.976**	−0.986**
TOM	−0.683*	−0.984**
CEC	−0.976**	−0.906**

*Shows the correlation reached P < 0.05, **shows the correlation reached P < 0.01. TOM, total organic matter; CEC, cation exchange capacity; bio-Cd, bioavailable Cd.

At the phylum level, the bio-Cd content in *P. notoginseng* planting soil was found to be positively correlated with the relative abundance of Acidobacteria (2018, 0.980^∗^; 2019, 0.520); by contrast, the bio-Cd content was found to be negatively correlated with the relative abundances of Proteobacteria (2018, −0.781^∗^; 2019, −0.925^∗^) and Chloroflexi (2018, −0.781^∗^; 2019, −0.967^∗^) in the soil bacteria (Table 4). The bio-Cd content was also found to be negatively correlated with Mortierellomycota in the fungi (2018, −0.732^∗^; 2019, −0.960^∗^), but positively correlated with Ascomycota in 2018 (0.781^∗^) and 2019 (0.913^∗^).

**TABLE 4:** Correlation analysis of bioavailable Cd content and soil microbial community in *P. notoginsen*g planting soil under different K_2_SO_4_ treatments.

2018	Bacterial	2018	Fungal

Community phylum	Bioavailable Cd content	Community phylum	Bioavailable Cd content
Proteobacteria	−0.781*	Ascomycota	0.781*
Acidobacteria	0.980*	Mortierellomycota	−0.732*
Chloroflexi	−0.781*	–	
Verrucomicrobia	0.781*	–	

**Community class**	**Bioavailable Cd content**	**Community class**	**Bioavailable Cd content**

Acidobacteriia	0.927**	Sordariomycetes	0.781*
Verrucomicrobiae	−0.781*	Mortierellomycetes	−0.732*
Deltaproteobacteria	−0.878**	Leotiomycetes	0.781*

**2019**	**Bacterial**	**2019**	**Fungal**

**Community phylum**	**Bioavailable Cd content**	**Community phylum**	**Bioavailable Cd content**

Proteobacteria	−0.925**	Ascomycota	0.913**
Acidobacteria	0.520	Mortierellomycota	−0.960**
Chloroflexi	−0.967**	Basidiomycota	−0.883*
Gemmatimonadete	0.921**	Rozellomycota	−0.983**
Actinobacteri	−0.924**	Glomeromycota	−0.963*

**Community class**	**Bioavailable Cd content**	**Community class**	**Bioavailable Cd content**

Acidobacteriia	0.945**	Sordariomycetes	0.920**
Holophagae	−0.976**	Mortierellomycetes	−0.960**
Alphaproteobacteria	−0.858**	Eurotiomycetes	−0.930**
Gemmatimonadete	0.914**	–	–
Gammaproteobacteria	−0.917**	–	–

*Shows the correlation reached P < 0.05, **shows the correlation reached P < 0.01.

At the class level, the bio-Cd content was found to be positively correlated with Acidobacteriia (2018, 0.927^∗∗^; 2019, 0.945^∗∗^), but negatively correlated with Verrucomicrobiae (2018, −0.781^∗^) and Gammaproteobacteria (2019, −0.917^∗∗^) in the soil bacteria. Moreover, the bio-Cd content was found to positively correlated with the relative abundance of Sordariomycetes (2018, 0.781^∗^; 2019, 0.920^∗∗^) and Leotiomycetes (2018, 0.781^∗^), but negatively correlated with Mortierellomycetes (2018, −0.732^∗^; 2019, −0.960^∗∗^) in the soil fungi.

## Discussion

The bio-Cd content represents the portion of Cd in the soil that can be absorbed and utilized by plant. The pH, TOM, and CEC have been identified as the main factors that affect the bioavailability of heavy metals in soil (Li and Song, 2003; Liang et al., 2013), and can be significantly regulated by fertilization. Agbede et al. (2010) found that the application of NPK mixture fertilizers can improve the pH and organic carbon content of yam (*Dioscorea rotundata Poir*) planting soil. K fertilizer can regulate the functional groups of acidic substances in tobacco planting soil and chelate heavy metal ions by adsorption and can thereby lowering the Cd activity and bio-Cd content in soil (Wu et al., 2012).

In the present study, pot experiments demonstrated that increases in the amount of applied K fertilizer significantly improved the pH, TOM, and CEC in *P. notoginseng* planting soil (Figure 2), resulting in a significant decrease in the bio-Cd content in the soil (Figure 3). The field experiments also verified the aforementioned results (Figures 5, 9). The results indicated that pH, TOM, and CEC reduction were ameliorated under the K fertilizer treatment, reducing the bio-Cd content in the planting soil and ultimately reducing the migration of Cd from the soil to *P. notoginseng*.

Fertilization can also affect the compositions, abundances and activities of soil microbial species. Jiang (2017) found that N fertilizer could change bacterial soil into fungal soil and decrease the biomass and abundance of soil microbial communities. Zhang and Xu (2014) demonstrated that K fertilizer (carbon enzyme K) could reduce the abundance and diversity of microbial communities in tomato planting soil. The application of K fertilizer can promote increases in soil nutrients and thereby lead to rapid increases in the abundances of microbial species that require large amounts of nutrients (richness groups) (Smith and Paul, 1990). Correspondingly, the application of K fertilizer can also reduce the abundances of microbial species that do not require large amounts of nutrients (Jia et al., 2004).

Essel et al. (2019) found a significant negative correlation between the abundances of bacteria, such as Holophagae, and pH in a 2-year spring wheat–pea rotation soil annually. In this study, the relative abundance of the class Acidobacteriia from the phylum Acidobacteria in *P. notoginseng* planting soil bacteria was found to be negatively correlated with the soil pH and CEC (Table 2). This pattern most likely resulted from the fact that most bacteria in Acidobacteria are acidophilic. However, increases in the application of K fertilizer inhibited the proliferation of the acidophilic population and thereby delayed decreases in the soil pH. Zhang et al. (2019) also indicated that the pH and TOM were significantly correlated with bacterial community abundance and that the pH was significantly correlated with fungal community composition, such as the abundance of Mortierellomycota.

The relative abundance of Mortierellomycota in *P. notoginseng* planting soil fungi was found to be positively correlated with the pH, TOM, and CEC (Table 1). This pattern can likely be attributed to the participation of Mortierellomycota in the mineralization of soil organic matter for the decomposition of crop residues into the soil and organic matter in organic fertilizers. Thus, the TOM content raised as the abundance of Mortierellomycota increased. Proteobacteria and Bacteroidetes belong to the richness groups, which can promote the mineralization of organic matter. Liu (2019) found that there are positive correlations of Proteobacteria and Bacteroidetes with the soil carbon availability. The present study found that the abundances of Proteobacteria and Bacteroidetes both increased as the amount of applied K fertilizer increased, which promoted the accumulation of the TOM (Table 1). This pattern shows that the abundances of Proteobacteria and Bacteroidetes of Fungi in the soil were promoted by K treatment, which facilitated the increases in the TOM.

Yao et al. (2019) determined that the bio-Cd content was significantly correlated with the diversity and abundance of microbial communities. In the present study, it was observed that the bio-Cd content was positively correlated with the relative abundance of the class Acidobacteriia from the phylum Acidobacteria, but negatively correlated with the Proteobacteria and Mortierellomycota in the soil (Table 4). Therefore, as the amount of K fertilizer applied increased, the relative abundances of the dominant soil microbes in the community changed, thereby mitigating reductions in the pH, TOM, and CEC in the soil. As a result, the bio-Cd content in the soil and Cd content in *P. notoginseng* were reduced (Figures 1, 4). According to traditional theories, K can change the bio-Cd content by altering the soil physical and chemical properties. However, the present study suggests another possibility: namely, that the effects of K on the bio-Cd content may be mediated by its effect on soil microorganisms, which, in turn, alter pH, TOM, and CEC.

The reduction of the Cd content by K may be achieved by (i) decreasing the bio-Cd content in soil via absorption via crops and by (ii) reducing the capability of crops to uptake Cd. Non-selective cation channels play a substantial role in root Cd uptake. This process is driven by the electrochemical gradient for Cd^2+^ on both sides of the plasma membrane. The membrane potential (between −70 and −90 mv) is often very close to the Nernst potential for K (available K, −70 mv); consequently, apoplast (soil) K^+^ availability is increased, leading to membrane depolarization. Thus, reduced Cd accumulation in plants may also be caused by the weaker electrical gradient across the plasma membrane. However, these hypotheses require further verification.

## Conclusion

A moderate K_2_SO_4_ treatment (pot experiment, 0.6 g⋅kg^–1^; field experiment, 300 kg⋅ha^–1^) provides the most optimal reduction of Cd accumulation in *P. notoginseng*. As the amount of applied K fertilizer increased, the relative abundances of Proteobacteria and Bacteroidetes increased, which promoted the accumulation of TOM; in addition, decreases in Acidobacteria alleviated the acidification of the soil. Such changes in these aforementioned soil microorganisms improved the pH, TOM, and CEC, which reduced the bio-Cd content in the soil and, in turn, the accumulation of Cd in the *P. notoginseng* roots was significantly reduced.

## Data Availability Statement

The datasets generated for this study can be found in the sequences were submitted to the SRA (Sequence Read Archive) at the National Center for Biotechnology Information (NCBI) under the accession number PRJNA626539 for 16S sequences (B1–B12) and ITS sequences (F1–F12).

## Author Contributions

YS was in charge of field experiment and pot experiment, and wrote the manuscript. LQ was in charge of determination of Cd content. LG was in charge of providing experimental ideas and revision the manuscript. JM was in charge of planting of Panax notoginseng. BS was in charge of harvesting of Panax notoginseng. RP was in charge of determination of soil physical and chemical properties. XO was in charge of determination of microbial diversity and population composition. CD was in charge of speciation of Cd in the soil and determination of Cd content. PL was in charge of statistical analysis. YY designed the whole experiment. XC was in charge of revision the manuscript. All authors contributed to the article and approved the submitted version.

## Conflict of Interest

The authors declare that the research was conducted in the absence of any commercial or financial relationships that could be construed as a potential conflict of interest.

## Abbreviations

Bio-Cd: Bioavailable Cd
CEC: Cation exchange capacity
F(Carb): Bound to carbonates fraction
F(EXC): Exchangeable fraction
F(Fe-MnOX): Bound to iron and manganese oxides fraction
F(OM): Bound to organic matter fraction
F(RES): Residual fraction
OTUs: Operational Taxonomic Units
RDA: Redundancy analysis
TOM: Total organic matter.
