# Supplementary figures and images for: K Fertilizers Reduce the Accumulation of Cd in Panax notoginseng (Burk.) F.H. by Improving the Quality of the Microbial Community
Source: Front Plant Sci. 2020 Jun 26;11:888. doi: 10.3389/fpls.2020.00888 (PMC7332963; doi:10.3389/fpls.2020.00888)

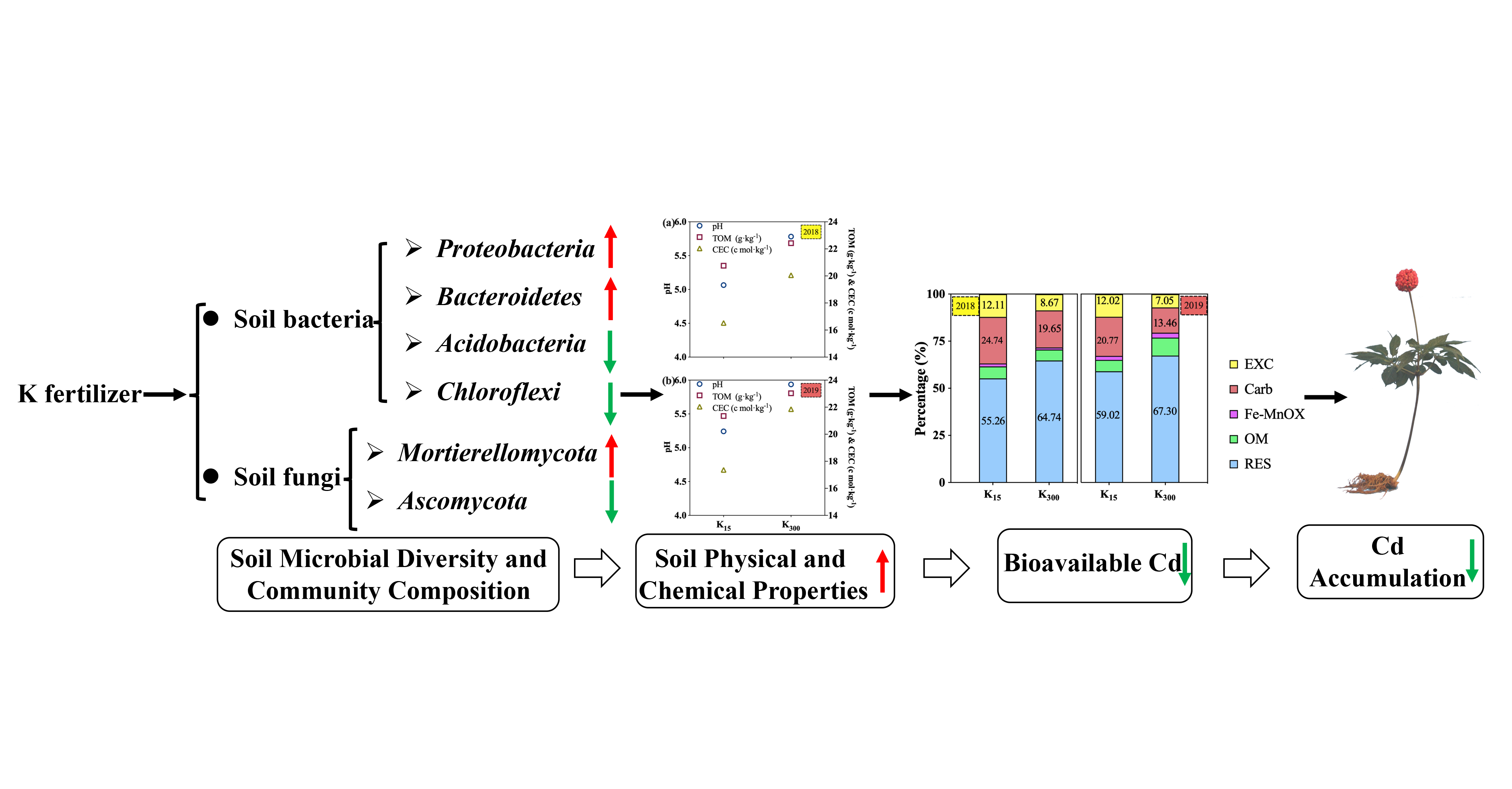

Supplement: Supplementary file 1 [file Image_1.TIF]
